# Management of infants born to women infected with hepatitis B in the military healthcare system

**DOI:** 10.1186/1756-0500-6-338

**Published:** 2013-08-28

**Authors:** Rebecca J Sainato, Elizabeth G Simmons, Dawn F Muench, Mark W Burnett, LoRanée Braun

**Affiliations:** 1 Department of Pediatrics, Tripler Army Medical Center, Honolulu, HI 96859-5000, USA; 2 Department of Pediatrics, Madigan Healthcare System, Tacoma, WA, USA

**Keywords:** Electronic health records, HBIG, Hepatitis B, Hepatitis B vaccines, Military, Newborns

## Abstract

**Background:**

Hepatitis B virus (HBV) is endemic worldwide. Given significant rates of infectivity, all infants born to Hepatitis B surface antigen positive mothers need to receive treatment at birth, immunization and post-vaccination serologic testing. However, not all infants complete these requirements.

**Findings:**

We performed a retrospective review of the management of infants born to Hepatitis B infected mothers at two large military hospitals in the United States that use a global electronic medical record to track patient results. We then compared these results to those recently published by the National Perinatal Hepatitis B Prevention Program (PHBPP), which does not include hospitals in the United States Military Healthcare System. Our results show that although all infants were managed appropriately at birth and immunization rates were very high, post vaccination follow-up testing rates were much lower than those seen in centers participating in the PHBPP. The rates of post vaccination serological testing were significantly higher for infants born to Hepatitis B e antigen positive mothers and those referred to a pediatric infectious disease specialist.

**Conclusions:**

Despite use of a global electronic medical record in the United States Military Healthcare System, management of HBV-exposed infants does not always follow recommended guidelines. These infants could benefit from a more systematic method of follow-up, similar to the PHBPP, to ensure HBV serologic testing is obtained after the vaccination series is complete.

## Findings

### Background

The estimated number of annual births to Hepatitis B surface antigen (HBsAg) positive women in the United States has been steadily increasing [1]. The incidence of perinatal HBV acquisition from HBsAg positive/Hepatitis B e antigen (HBeAg) positive mothers is greater than 90% [2], and >50% amongst unvaccinated but chronically exposed infants and toddlers [3], but with proper treatment this rate may be reduced to 10-15%. Although much lower, there is also a significant risk of perinatal HBV acquisition from HBsAg positive/HBeAg negative mothers. The National Perinatal Hepatitis B Prevention Program (PHBPP) recently published their data analyzing the effectiveness of their interventions [1]. We conducted this study to see how the United States Military Healthcare System (MHS), with its unified global electronic medical record (EMR), compares to the PHBPP in managing newborns who are at risk of acquiring HBV.

### Materials and methods

We performed a retrospective review of the management of infants born to Hepatitis B infected mothers at two of the largest military treatment facilities: Tripler Army Medical Center in Honolulu, Hawaii and Madigan Healthcare System in Tacoma, Washington. Cases were identified between September 2005 and September 2009 by searching for all women who were coded in our medical system as being both pregnant with a normal delivery and also having any type of hepatitis. This initial list was narrowed to women with hepatitis B by reviewing laboratory results of each woman meeting these criteria and selecting only those who were HBsAg positive. We further defined our population’s level of infectivity by evaluating for the presence of HBeAg, but included patients who were both HBeAg positive and negative in our analysis.

Each inpatient delivery record was then reviewed to evaluate the exposed newborn’s management and determine whether the infant had received HBIG (Hepatitis B immunoglobulin) and HBV vaccine as recommended on the first day of life. Pharmacy records were also queried for a list of all infants to whom HBIG was dispensed.

These infants’ outpatient courses were then analyzed to determine whether they went on to complete their HBV vaccination series by 12 months (2 additional vaccines) and had subsequent serologic testing by 18 months (HBsAg and anti-HBs). Only patients who demonstrated ongoing care within the MHS, characterized by at least four documented well baby appointments over the first year of life and at least one encounter after 15 months, were included in the study group.

In addition, we determined if exposure to HBV was documented within the outpatient record and if the child had seen our pediatric infectious disease (ID) specialists regarding the exposure. These variables were compared by two-sided Fisher’s exact test to determine the association with completing the recommended vaccine series and follow-up serologic testing.

### Ethics

The study was approved by the Institutional Review Boards at both Tripler Army Medical Center and the Madigan Healthcare System.

### Results

Both medical centers had very similar results. Cumulatively, from September 2005 to September 2009 there were sixty-seven infants born to sixty-three HBsAg positive mothers; fourteen (22%) of these mothers were HBeAg positive. Of the sixty infants born to HBsAg positive mothers whose inpatient medical records were available to review, 100% were given HBIG and their initial hepatitis B vaccination within 1 day of birth. Fifty-one children were subsequently followed in the MHS. 92% completed their vaccine series by 12 months, but only 10% received post-vaccination testing by 18 months. In comparison, 94% of infants in the PHBPP received recommended management at birth, and only 78% completed their vaccine series by 12 months. However, 56% of those infants received post-vaccination testing by 18 months of age [1].

18% of the followed infants in this study were born to mothers who were also HBeAg positive. Of those, 89% completed their vaccination series and 33% were followed up at 18 months for post-vaccination testing.

HBV exposure of the infants who were followed in our system was never mentioned in more than half of their outpatient medical records and documentation of the exposure was not associated with serological testing completion rates. However, the rates of post vaccination serological testing were significantly higher for infants born to HBeAg positive mothers and those referred to a pediatric ID specialist despite national guideline recommendations of follow-up testing for all exposed infants (HBeAg positive and negative) (Table 1).

**Table 1:** Completion rates for recommended vaccinations and post-vaccination serological testing

**Population**	**n**	**Completed vaccination series**		**Post vaccination serology**	
		**Percentage**	***P *value**	**Percentage**	***P *value**
**Maternal HBsAg + infants**	51	92%		10%	
**Maternal HBsAg + infants with Ped ID referral**	9	89%	(0.552)	**33%**	**(0.033)**
Maternal HBsAg + infants whose exposure was documented*	24	92%	(1.00)	13%	(0.656)
**Maternal HBsAg+ / HBeAg + infants**	9	89%	(0.552)	**33%**	**(0.033)**
**Maternal HBsAg+ / HBeAg + infants with Ped ID referral**	4	100%	(1.00)	**75%**	**(0.048)**
Maternal HBsAg+ / HBeAg + infants whose exposure was documented*	7	100%	(0.222)	43%	(0.500)

^*^ Exposure documented within the infant’s electronic medical record.

Exposure to HBV infection and evaluation by a pediatric infectious disease specialist were compared using two-sided Fisher’s exact test to determine the association with completing the recommended vaccine series and completing follow-up serologic testing. Both patients who were HBeAg positive and all patients (HBsAg positive and HBSAg positive/HBeAg positive) who saw an infectious disease specialist were more likely to have post-vaccination serologies checked. There were no statistically significant differences in vaccination rates amongst the different groups.

### Discussion

This small study examines the care of HBV-exposed infants within the United States MHS, which in contrast to most hospital systems, has a unified global EMR. Overall compliance with initial newborn management and vaccination series completion at these two major military medical centers far exceeded the national average by comparison with the PHBPP, but most of our infants did not have serologic testing by 18 months. Although this could introduce a small systematic bias, we used our schedule of receiving 3 vaccines by 12 months and obtaining serologic testing by 18 months because this allowed us to compare our results to the PHBPP who used the same model. In addition, the American Academy of Pediatrics Committee on Infection Diseases recommends that infants born to HBsAg-positive women should be tested for anti-HBs and HBsAg after completion of the immunization series, at 9 to 18 months of age [4]. The highest rates of follow-up testing among our population occurred in infants referred to a pediatric ID specialist, which suggests that in the absence of a systematic method, subspecialty referral facilitated the highest testing rates.

A limitation of our study is that there is now data to support the use of lamivudine in pregnancy to reduce viral load and thereby limit perinatal transmission [5]. Treatment with lamivudine was not standard of care at our institutions for the cases analyzed from 2005–2009 and has only been adopted more recently. While this may reduce transmission rates from mother to the child, this does not change current vaccination or follow-up serological testing recommendations of the offspring nor alter the validity of our findings.

Provision of health care within the MHS is marked by many unique challenges in managing infants born to HBsAg positive mothers. There is frequent movement of families, often internationally. Additionally, within the MHS the inpatient and outpatient medical records are not connected, so management plans documented at birth are not routinely visible to outpatient clinics. An indirect outcome of this study was identification that HBV exposure is often not documented within the outpatient medical record. Nevertheless, the vast majority of our patients are still identified at birth and vaccinated on time, evidencing that the challenges of a transient population may be overcome with the help of a global EMR. Instead, the primary issue needing improvement is chronic management of a unique risk factor in the setting of poor continuity of care, which has proven difficult to overcome by use of an EMR alone. We are not aware of other published reports to-date that use an EMR to ensure follow-up of HBV exposed infants. Our data shows that HBV-exposed infants within the MHS would benefit from a more systematic method of follow-up, similar to the PHBPP, to ensure follow-up HBV serologic testing is obtained as recommended.

## Abbreviations

EMR: Electronic medical record; HBeAg: Hepatitis B e antigen; HBsAg: Hepatitis B surface antigen; HBIG: Hepatitis B immunoglobulin; HBV: Hepatitis B virus; ID: Infectious disease; MHS: Military healthcare system; PHBPP: National perinatal hepatitis B prevention program.

## Competing interests

The authors declare that they have no competing interests.

## Authors’ contributions

RJS and LB conceived and designed the study. RJS, EGS, DFM, MWB and LB contributed significantly in reviewing the proposal, data collection, analysis, and interpretation of the data. RJS and EGS drafted the manuscript. All authors read and approved the final manuscript.
